# Hexarelin treatment preserves myocardial function and reduces cardiac fibrosis in a mouse model of acute myocardial infarction

**DOI:** 10.14814/phy2.13699

**Published:** 2018-05-13

**Authors:** Hayley McDonald, Jason Peart, Nyoman Kurniawan, Graham Galloway, Simon Royce, Chrishan S. Samuel, Chen Chen

**Affiliations:** ^1^ School of Biomedical Science University of Queensland Brisbane Australia; ^2^ Menzies Health Institute of Queensland Griffith University Gold Coast Australia; ^3^ Centre for Advanced Imaging University of Queensland Brisbane Australia; ^4^ Cardiovascular Disease Program Biomedical Discovery Institute and Department of Pharmacology Monash University Victoria Australia; ^5^ Central Clinical School Monash University Victoria Australia

**Keywords:** Fibrosis, heart failure, ischemia, myocardial infarction, remodeling

## Abstract

Ischemic heart disease (IHD) is a leading cause of morbidity and mortality worldwide. Growth hormone secretagogues (GHS) have been shown to improve cardiac function in models of IHD. This study determined whether hexarelin (HEX), a synthetic GHS, preserves cardiac function and morphology in a mouse model of myocardial infarction (MI). MI was induced by ligation of the left descending coronary artery in C57BL/6J mice followed by vehicle (VEH;* n* = 10) or HEX (0.3 mg/kg/day; *n* = 11) administration for 21 days. MI‐injured and sham mice (treated with VEH;* n* = 6 or HEX;* n* = 5) underwent magnetic resonance imaging for measurement of left ventricular (LV) function, mass and infarct size at 24 h and 14 days post‐MI. MI‐HEX mice displayed a significant improvement (*P *< 0.05) in LV function compared with MI‐VEH mice after 14 days treatment. A significant decrease in LV mass, interstitial collagen and collagen concentration was demonstrated with chronic HEX treatment (for 21 days), accompanied by a decrease in TGF‐*β*1 expression, myofibroblast differentiation and an increase in collagen‐degrading MMP‐13 expression levels. Furthermore, heart rate variability analysis demonstrated that HEX treatment shifted the balance of autonomic nervous activity toward a parasympathetic predominance and sympathetic downregulation. This was combined with a HEX‐dependent decrease in troponin‐I, IL‐1*β* and TNF‐*α* levels suggestive of amelioration of cardiomyocyte injury. These results demonstrate that GHS may preserve ventricular function, reduce inflammation and favorably remodel the process of fibrotic healing in a mouse model of MI and hold the potential for translational application to patients suffering from MI.

## Introduction

Ischemic heart disease (IHD) is a leading cause of morbidity and mortality worldwide (Suvarna 2013; Hausenloy and Yellon 2013). A variety of risk factors contribute to the development of heart failure (HF) post myocardial infarction (MI) and ultimately cumulate in structural changes within the myocardium leading to left ventricular (LV) dysfunction. A key event in the progression of HF is the pathological remodeling of the ventricle secondary to cardiac fibrosis. Cardiac fibrosis has been identified as both a primary and causal driver of disease and has become an important therapeutic target in HF patients (Dixon et al. 2015). Stiff fibrotic tissue affects myocardial electrical transmission leading to arrhythmias, contributes to diastolic and systolic dysfunction and can ultimately result in HF (Dixon et al. 2015). The mechanisms that underpin the stimulation of cardiac fibrosis are not fully understood and interventions targeting excessive fibrosis as a result of MI are of major therapeutic importance (Samuel et al. 2011; Segura et al. 2014).

Growth hormone secretagogues (GHS) have been recently identified as compounds that stimulate growth hormone (GH) release from the pituitary gland through the G protein‐coupled receptor (GHS‐R) expressed mainly within the pituitary and hypothalamus (Baldanzi et al. 2002; Kojima & Kangawa, 2005). The endogenous ligand, ghrelin, was originally isolated from the stomach wall in 1999 (Kojima et al. 1999). GHS have many functions other than the control of GH‐release. Ghrelin plays a major role in the regulation of systemic metabolism and has been shown to exert a number of central and peripheral actions such as regulation of food intake, control of energy balance, glucose metabolism, and insulin release, and stimulation of gastric acid secretion and motility (Kojima and Kangawa 2005; Cheng et al. 2010). The GHS‐R is expressed in many peripheral organs including the stomach, intestine (Date et al. 2000), pancreas (Guan et al. 1997), kidney (Mori et al. 2000), heart and aorta (Nagaya et al. 2001). GHS have been demonstrated to have several cardiovascular effects suggested by the presence of GHS‐R on blood vessels and ventricular tissue (Kishimoto et al. 2009). Hexarelin (HEX), a synthetic hexapeptide belonging to the GHS family has been reported to feature cardiovascular activity and is recognized to be both chemically more stable and functionally more potent than ghrelin (Deghenghi 1998; Mao et al. 2013). The GHS‐R appears to mediate the action of HEX (Mao et al. 2013, 2014a). The antifibrotic effects of GHS have been investigated with promising results in various models of cardiac injury such as doxorubicin cardiotoxicity isoproterenol administration, myocardial infarction, and spontaneous or diabetes‐associated hypertension (Angelino et al. 2015).

This study was designed to investigate the therapeutic effects of HEX on myocardial function and cardiac fibrosis using a murine model of MI. Here, we demonstrate a preservation of systolic and diastolic function measured by cardiac magnetic resonance imaging (cMRI) and a HEX‐dependent attenuation of cardiac fibrosis. We propose that HEX prevents cardiac fibroblasts from acquiring a proinflammatory and fibrogenic phenotype, preventing excessive collagen synthesis and cardiac fibrosis. This may be mediated by HEX's influence on inflammation and the autonomic nervous system evidenced by a reduction in inflammatory cytokines, parasympathetic predominance and sympathetic downregulation, respectively. Thus, the findings of this study indicate that HEX may target fibrotic pathways and preserve LV function in a mouse model of MI.

## Materials and Methods

### Animal models of MI

#### Ethical approval

All experiments were approved by the Animal Ethics Committee of the University of Queensland and were performed in accordance to national guidelines (Ethics number SBMS/200/13/NHMRC). The investigators understand and comply with the ethical principles of the Journal of Physiology.

MI was induced in 12–14 week old male C57BL/6JArc inbred mice (weight 25–30 g) (*n* = 21) (JAX stock number: 000664) by permanent ligation of the left anterior descending coronary artery (LAD). Mice were anesthetized using a combination of medetomidine (1 mg/kg) and ketamine (75 mg/kg) administered by intraperitoneal injection, intubated and supported by a small animal ventilator (Harvard Apparatus) with tidal volume and respiratory rate calculated based on body weight (Tarnavski et al. 2004). Anesthetic depth was monitored by assessment of the pedal reflex and heart rate by electrocardiographic (ECG) monitoring. A left‐sided thoracotomy was performed and the LAD was ligated 2 mm below the left auricular appendage using 7‐0 Prolene. Successful occlusion was confirmed by visualization of a pallor region in combination with characteristic ECG changes. Sham‐operated mice (*n* = 11) underwent the same procedure excluding ligation of the LAD. The chest was closed using 6‐0 polydioxanone and the musculature and cutaneous tissues closed using a 5‐0 nonabsorbable suture.

After completion of the surgery the mice were administered atipamezole (1 mg/kg subcutaneous (SC)), carprofen analgesia (5 mg/kg SC) and this was continued once daily for 48 h postprocedure. A 0.5 mL bolus of saline was administered SC on recovery. The mice were recovered in an oxygen‐ and heat‐supplemented environment and then moved to their standard housing consisting of a temperature‐ and heat‐ controlled environment with 12‐hour light/dark cycles and provided with free access to food and water ad libitum where they remained until the completion of the study.

### Hexarelin and vehicle administration

HEX (0.3 mg/kg/day; *n* = 11) or vehicle (VEH; *n* = 10) was administered to each mouse SC within a 15‐min period prior to LAD ligation. Similarly, mice undergoing the sham procedure received either VEH (*n* = 6) or HEX (*n* = 5) treatment. All mice received their respective treatments once daily throughout the 21‐day study period. The HEX dose was chosen based on similar studies by Mao et al. (2013, 2014b).

### Cardiac magnetic resonance imaging

cMRI was performed at both 24 h and 14 days post‐LAD ligation or sham procedure in all animals. Mice were imaged using a 30‐cm‐diameter horizontal bore Bruker Biospec 9.4 Tesla (T) small animal MRI scanner equipped with a BGA 12S HP 660 mT/m gradient set. MRI data were acquired with an 86 mm i.d. quadrature transmit coil and a 2 × 2 phase array receive coil, running Paravision 5.1. (Bruker Biospin, Ettlingen, Germany).

#### Animal preparation

Anesthesia was induced in an induction chamber using 5% isoflurane in 100% medical grade oxygen with a flow rate of 1 L/min. The mouse was positioned in a purpose‐built cradle (Bruker, Germany) and maintained with 1.5–2% isoflurane in 1–2 L/min oxygen via a nose cone. Core temperature was monitored using a rectal probe and maintained with a warm water circulation system incorporated into the animal bed. A SAII Monitoring system (Small Animal Instruments, NY, USA) was used to measure ECG, using a 3‐lead system with surface Ag–AgCl electrodes and respiration was monitored with a pressure transducer, from which a respiratory gating signal could be derived.

#### cMRI protocol

Gadopententate dimeglumine (Gd‐DTPA) (0.3 mmol/kg Magnevist, Bayer, Germany) was administered by intravenous (IV) infusion into the lateral tail vein of the mouse. Following standard prescan calibration, 2‐ and 4‐chamber view scout scans were acquired, from which a single mid‐cavity short‐axis slice was planned. Cine imaging was performed with a retrospectively triggered (self‐gated) INTRAGATE gradient‐echo sequence (Bovens et al. 2011), with the following parameters: TR = 5.6 ms, TE = 2.6 ms, flip angle = 10 degrees, number of movie frames = 20, slice thickness = 1 mm, matrix = 512 × 512, field‐of‐view (FOV) = 4 × 4 cm^2^. This resulted in 78 × 78 μm in‐plane resolution, with ~5 min acquisition per slice. Seven to nine short‐axis slices with no slice gap were acquired to cover the heart from apex to base. Late gadolinium enhancement (LGE) images in the slice locations described above were acquired 10–15 min after IV Gd‐DTPA and assessed for LGE.

#### Determination of cardiac functional parameters and LV mass

MRI images, in DICOM format, were processed with Osirix (Rosset et al. 2004) software. The end‐diastolic and end systolic phases were identified on a slice‐by‐slice basis and both the endocardial and epicardial borders were traced. The LV end systolic volume (ESV), end diastolic volume (EDV), stroke volume (SV), and ejection fraction (EF) were computed from the traced borders and LV mass was obtained by multiplying the volume by the specific gravity of 1.05 g/cm^3^ (Bohl et al. 2009).

#### Determination of infarct volume

The LGE images were windowed to maximize the signal from the hyperintense region and null that from the nonenhanced region. Manual planimetry was performed on each slice and the hyperintense region and total LV myocardial area was calculated. Slice hyperintense areas were multiplied by the slice thickness and summed to calculate the infarct volume as a percentage of LV myocardial volume.

#### Measurement of the chronic infarct: midline‐length measurement

In addition to infarct volume, the infarct length was quantified, 14 days post‐MI, using an alternative cMRI approach described as a “midline‐length measurement.” This technique was modified from Takagawa et al. (2007). Briefly, the *midline infarct length* was determined by measuring the midline length of the infarct that included 50% of the whole thickness of the myocardial wall. The LV myocardial midline was measured as a *midline circumference*. All measurements were taken in diastole. Infarct size was calculated by dividing the sum of midline infarct length from all sections by the sum of the midline circumference from all sections and multiplying by 100.

#### Post mortem measurement of Infarct size

At the endpoint of the study (at day 21 post‐MI) mice were killed by cervical dislocation. In a subset of mice (HEX (*n* = 5), VEH (*n* = 4)), the hearts were rapidly excised, trimmed of extracardiac tissue and flushed with saline to remove any remaining blood. Hearts were fixed in OCT compound at −20°C for 15 min and transected into 6–8 short‐axis slices of 1 mm thickness using a Zivic Mouse Heart Slicer Matrix (Zivic Instruments). Slices were thawed and immersed in 1% triphenyl tetrazolium chloride (TTC) in PBS at 37°C for 20 min while being constantly agitated. The slices were fixed in 10% neutral‐buffered formalin overnight and each slice photographed on each side with a digital camera mounted on a stereomicroscope (Nikon C51). Manual planimetry of viable and nonviable tissue was performed using ImageJ (Schneider et al. 2012). Specifically, the total LV area and MI were manually traced for each image and MI size was determined as a percentage of LV mass.

### Histology and morphometric analysis

Serial paraffin‐embedded LV sections from each group of mice studied were stained with 0.1% picrosirius red to detect interstitial collagen deposition. Additional serial LV sections from each corresponding group of mice studied were immunohistochemically stained for selected markers associated with collagen turnover; utilizing polyclonal antibodies to transforming growth factor (TGF)‐*β*1 (sc‐146; 1:200 dilution; Santa Cruz Biotechnology Inc., Santa Cruz, CA, USA) and matrix metalloproteinase (MMP)‐13 (the predominant collagenase in mice; ab75606; 1:100 dilution; Abcam; Redfern, NSW, Australia); or a monoclonal antibody to *α*‐smooth muscle actin (*α*‐SMA; a marker of myofibroblast differentiation; M0851; 1:250 dilution; DAKO Antibodies, Carpinteria, CA, USA). Detection of primary antibody staining was detected using DAKO Envision antirabbit or ARK biotinylation kits, respectively, and 3,3‐diaminobenzidine. Morphometric analysis of picrosirius red‐ and immunohistochemically stained sections was performed using Aperio software (Leica Biosystems, North Ryde, NSW, Australia) on whole‐tissue sections per mouse. In each case, the percentage staining of each marker analyzed per section was derived and expressed as the fold changes relative to the SHAM‐VEH group, which was expressed as 1.

### Hydroxyproline analysis

Equivalent frozen portions of LV tissue from each animal studied were lyophilized to dry weight measurements before being hydrolyzed in 6 mol/L hydrochloric acid for determination of their hydroxyproline content, as described previously (Samuel 2009). Hydroxyproline values were converted to collagen content by multiplying by a factor of 6.94 (based on hydroxyproline representing approximately 14.4% of the amino acid composition of collagen in most mammalian tissues (Gallop and Paz 1975), further expressed as a percentage of the tissue dry weight (to yield collagen concentration), and finally expressed as the relative change, compared to the SHAM‐VEH group (which was expressed as 1).

### Heart rate variability analysis

Heart rate variability (HRV) analysis was performed 21 days after MI or sham procedure. ECG signals were recorded using a physiological analyzing system (Bio Amp, AD Instruments, CA, USA). Mice were anaesthetized with isoflurane and ECG signals were recorded for a minimum of 20 min after heart rate stabilization. Using a technique described in (Thireau et al. 2008); autonomic nervous system function was examined by power spectral analysis of HRV (LabChart Pro 7.0, ADInstruments, Australia). Heart rate was used to generate a power spectral density curve using a fast Fourier transformation. The area under the curve was calculated for the very‐low‐frequency (VLF: 0–0.15 Hz), low‐frequency (LF: 0.15–1.5 Hz), and high‐frequency (HFr: 1.5–5 Hz) band, based on previous studies (Thireau et al. 2008). From these, the parameters: LF, HFr, normalized LF power (nLF), normalized HF power (nHFr), and ratio of LF to HFr power (LF/HFr) were calculated as described in (Mao et al. 2012).

### Cytokine and troponin‐I determination

Blood samples were collected from mice at both 24 h and 21 days after the procedure. The blood was allowed to clot appropriately and samples were centrifuged for serum removal and stored at −80°C until assayed. The serum concentrations of troponin‐I (cTnI), interleukin (IL)‐1*β*, IL‐6, and tumor necrosis factor (TNF)‐*α* were measured using a MILLIPLEX® map assay according to the manufacturer's instructions (Merck Millipore).

### Statistical analysis

Data expressed as the mean ± SEM or relative mean ± SEM. Statistical analysis was performed using GraphPad Prism 7. Differences between groups were analyzed by one‐way ANOVA using either a Bonferroni or Newman Keuls post hoc test to allow for multiple comparisons between groups. Unpaired *t*‐test was applied to comparisons between two groups. *P *< 0.05 was considered statistically significant.

## Results

### cMRI measurement of cardiac function

#### LVEF, EDV, ESV

**Figure 1:**
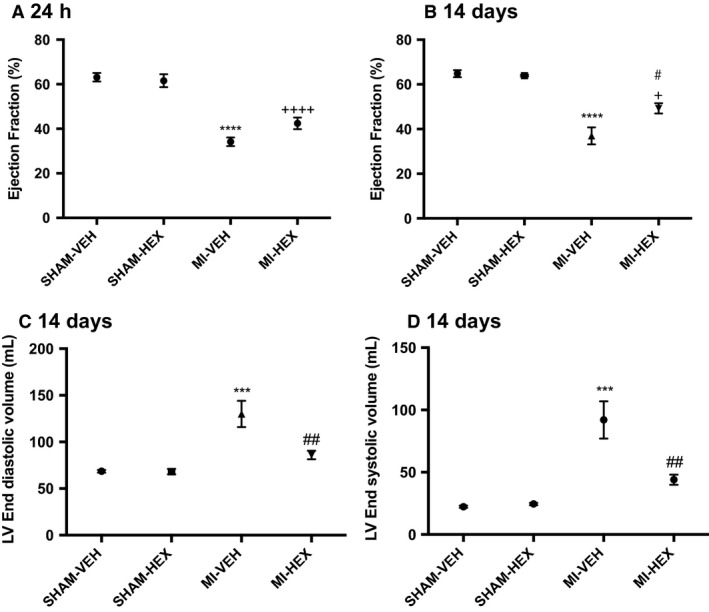
Cardiac functional parameters measured by cMRI. Ejection fraction (%), LV end‐diastolic volume, LV end‐systolic volume measured post‐MI/sham procedure with or without HEX treatment. (A). EF 24 h postprocedure, (SHAM‐VEH (*n* = 5), SHAM‐HEX (5), MI‐VEH (7), MI‐HEX (7)); (B) EF 14 days postprocedure (*n* = 6/5/10/11); (C) LV EDV 14 days postprocedure (*n* = 6/5/10/11); (D) LV ESV 14 days postprocedure (*n* = 6/5/10/11). Data in all figures expressed as mean ± SEM; ***P <* *0.001, ****P <* *0.0001, versus SHAM‐VEH group; ^#^ *P *<* *0.05, ^##^ *P *<* *0.01, versus MI‐VEH group; ^+^ *P *<* *0.05, ^++++^ *P*< 0.0001, versus SHAM‐HEX group. cMRI, cardiac magnetic resonance imaging; EDV, end diastolic volume; ESV, end systolic volume.

There was a significant reduction in EF in MI‐VEH and MI‐HEX mice 24 h post LAD occlusion. However, a trend toward a preservation of EF was observed in the MI‐HEX group compared with the MI‐VEH group (Fig. 1A). After 14 days treatment, HEX treatment significantly improved EF compared to MI‐VEH mice (HEX: 49.25% ± SEM 2.3; VEH: 36.96% ± SEM 3.82) (Fig. 1B).

The MI‐VEH group showed a significant increase in EDV 24 h post‐MI. These changes were completely reversed with 14 days of HEX treatment (Fig. 1C). LV ESV was also significantly elevated after 14 days in the MI‐VEH group, which again was entirely reversed with HEX treatment (Fig. 1D). These results suggested that HEX treatment may preserve both systolic and diastolic function post‐MI.

### Infarct assessment

**Figure 2:**
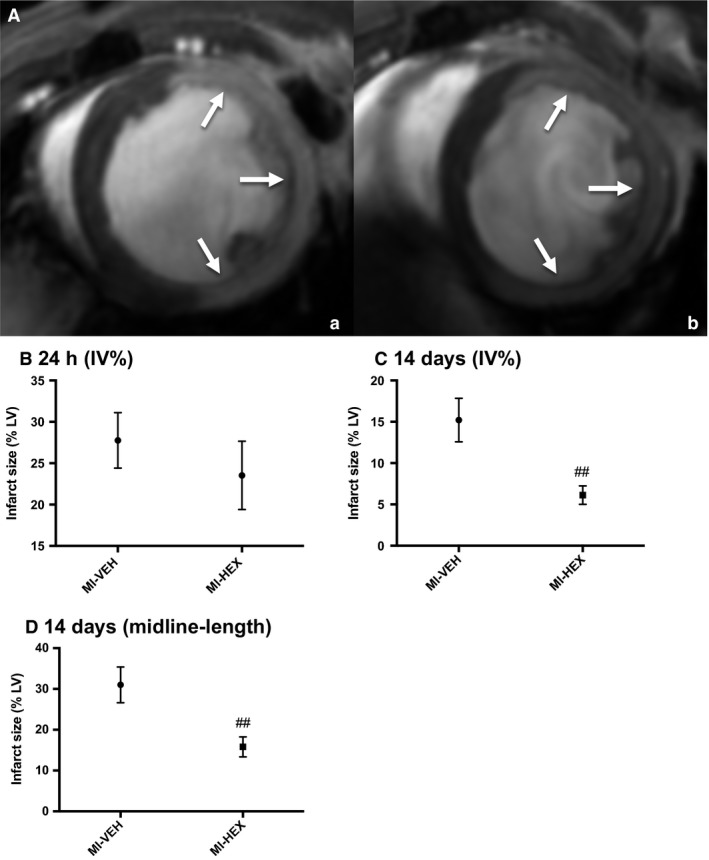
Infarct size measured by cMRI. (A). Representative LGE images acquired using a T_1_‐weighted INTRAGATE gradient‐echo sequence 24 h post LAD ligation. The hyperintense region represents the infarcted tissue (indicated by white arrows); (a). HEX‐treated (b). VEH‐treated. (B) Myocardial infarct size assessed 24 h post‐MI (IV%), (*n* = 7/7). (C) Myocardial infarct size 14 days post‐MI, infarct size assessed via IV% approach, or (D) via midline‐length approach, (*n* = 10/10). ^##^ *P *< 0.01 versus MI‐VEH group. IV%, Infarct volume %.

Infarct volume was determined using cMRI and manual planimetry to calculate infarct volume as a percentage of LV myocardial volume (IV%) after 24 h. Both treatment groups exhibited observable infarction after 24 h (Fig. 2Aa,b), there was no significant difference in IV% between MI‐HEX and MI‐VEH‐treated groups at this time point (Fig. 2B). This was confirmed by TTC staining in a subset of animal's postmortem.

Following 14 days of permanent LAD ligation, VEH‐ and HEX‐treated mice exhibited observable myocardial infarction as assessed by LGE. Infarct volume was significantly reduced following HEX treatment (HEX 6.13 ± 1.11; VEH: 15.21 ± SEM 2.63) (Fig. 2C). Similarly, when infarct size was assessed using the midline‐length measurement approach using cMRI, a significant reduction in infarct midline‐length was observed following HEX treatment (Fig. 2D). The midline‐length measurement for MI‐VEH mice was twofold greater than that assessed by the volume approach. In chronic infarcts, the length‐based approach has been demonstrated to measure the extent to which the infarct scar radially covers the wall of the LV, without being influenced by thinning of the wall, thus this is likely to explain the different values obtained by the two measurement approaches. The midline‐length approach may represent a superior measure to assess chronic infarcts (Takagawa et al. 2007).

### LV size

**Figure 3:**
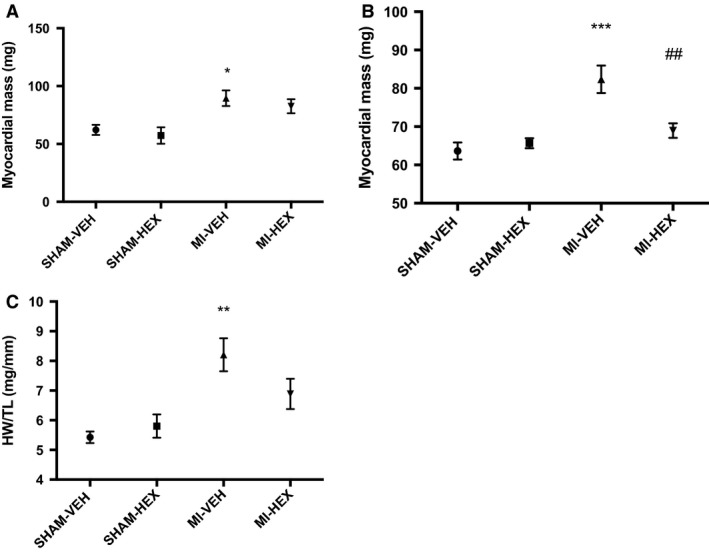
Assessment of LV mass and heart weight. LV myocardial mass (mg) assessed by cMRI after: (A) 24 h, (SHAM‐VEH/HEX (5), MI‐VEH/HEX (7)); (B) 14 days post‐MI/sham procedure with or without HEX treatment, (*n* = 6/5/10/11). (C) Heart weight compared 21 days postprocedure. Tibial length (TL) was used as an adjustment factor, (*n* = 6/5/5/6). *P < 0.05, **P < 0.01, ***P < 0.001, versus SHAM‐VEH group; ^##^ *P *< 0.01, versus MI‐VEH group. TL, tibial length.

LV myocardial mass was determined using cMRI 24 h and 14 days postprocedure. There was a significant difference in mass between MI‐VEH and the respective SHAM‐VEH group after 24 h, whereas this was not apparent within the MI‐HEX group. At this acute time point, there is unlikely to be any significant effect on remodeling but it is thought that these changes may reflect differences in inflammation and/or edema (Fig. 3A). Cardiac mass was significantly elevated after 14 days in the MI‐VEH group. This increase was entirely reversed by HEX treatment (Fig. 3B).

To further evaluate cardiac hypertrophy, heart weight (HW) was compared 21 days post procedure. Tibial length (TL) was used as an adjustment factor as body weight and growth tendencies were found to depend on whether MI or sham operation was performed (Mao et al. 2013). HW/TL ratios did not differ between the two sham groups. The MI‐VEH HW/TL was significantly higher than the SHAM‐VEH group. There was a trend toward a decreased HW/TL ratio in the MI‐HEX group compared with the MI‐VEH group (Fig. 3C). Thus, the HW/TL results were consistent with our cMRI myocardial mass data.

### The effect of HEX on MI‐induced cardiac remodeling

To determine the effect of HEX on LV fibrosis in MI, mice were treated with HEX or VEH daily for 21 days.

#### LV collagen deposition

**Figure 4:**
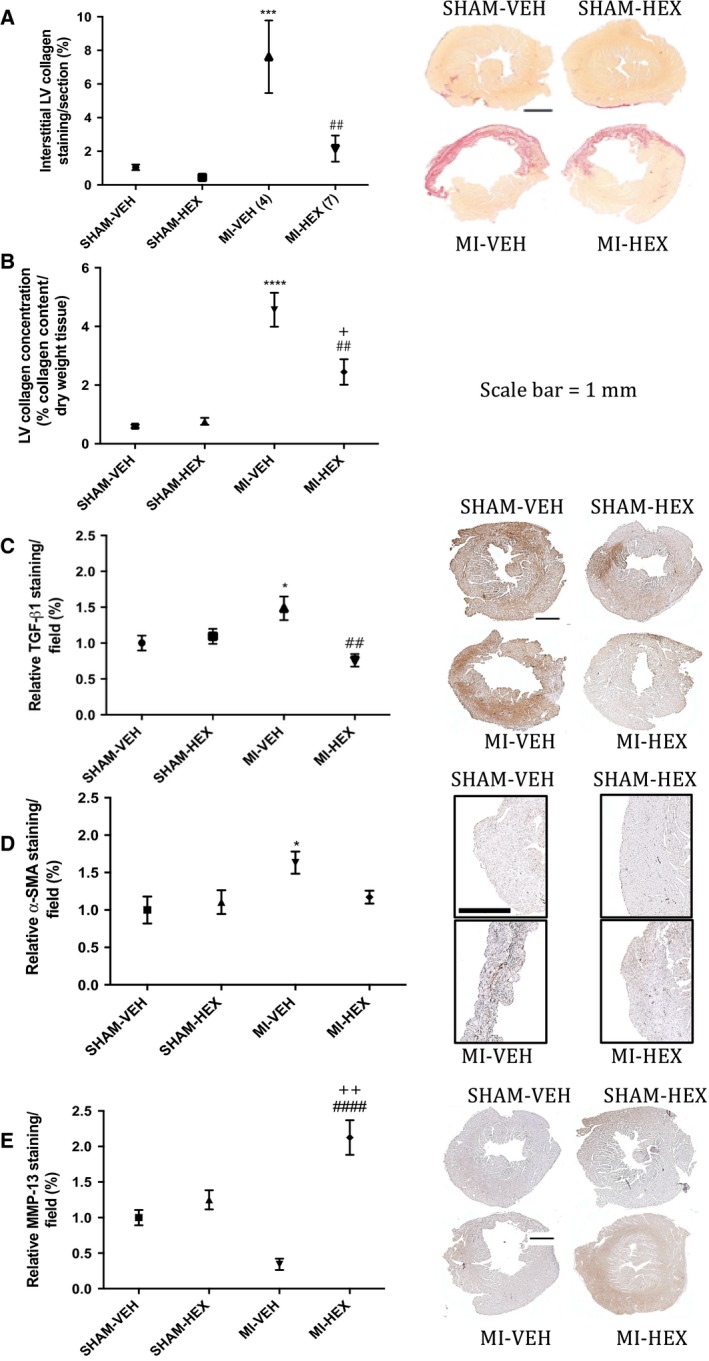
Histology and morphometric analysis. (A) Interstitial LV collagen content measured by picrosirus red staining, (*n* = 6/5/6/7); (B) LV collagen concentration, measured by hydroxyproline analysis, (*n* = 6/5/4/7); (C) TGF‐*β*1 (6/5/5/6), (D) *α*‐SMA (5/6/5/7), and (E) MMP‐13 immunohistochemistry staining (*n* = 6/5/5/6) measured 21 days post‐MI/sham procedure with or without HEX treatment. The right column displays representative sections from each treatment group corresponding to the selected markers and methods described above. *P < 0.05, ***P < 0.001, ****P < 0.0001, versus SHAM‐VEH group; ^##^ *P *< 0.01, ^####^ *P *< 0.0001, versus MI‐VEH group; ^+^ *P *< 0.05, ^++^ *P *< 0.05; versus SHAM‐HEX group.

Picrosirus red staining of the myocardium was used to detected changes in interstitial collagen deposition and was markedly increased in the MI‐VEH group (7.62% of the LV area stained ± SEM 2.15) compared to that from the SHAM‐VEH group (1.05% ± SEM 0.17). The presence of HEX (MI‐HEX) was seen to almost completely reverse the aberrant postinfarct interstitial collagen deposition measured compared with the MI‐VEH group (2.15% ± SEM 0.78; Fig. 4A). Left ventricular collagen concentration (% collagen content per dry weight tissue) was extrapolated from hydroxyproline analysis of tissue samples, and was also significantly higher in MI‐VEH mice (4.57% ± SEM 0.58) compared to their SHAM‐VEH counterparts (0.60 ± SEM 0.06). Chronic treatment with HEX resulted in a ~53% reduction (2.45% ± SEM 0.44) in LV collagen concentration (Fig. 4B).

#### TGF‐*β*1, *α*‐SMA, and MMP‐13

LV TGF‐*β*1 (Fig. 4C) and *α*‐SMA‐associated myofibroblast (Fig. 4D) immunostaining was significantly increased (by ~49% and ~63%, respectively); whereas LV MMP‐13 staining (Fig. 4E) was significantly reduced (by ~66%) in MI‐VEH mice compared to that measured in SHAM‐VEH mice.

HEX (MI‐HEX) therapy normalized the aberrant MI‐induced increase in TGF‐*β*1 (Fig. 4C) and *α*‐SMA (Fig. 4D) expression, and increased MMP‐13 levels to sixfold of that measured in the MI‐VEH group (Fig. 4E). These results suggested that HEX had a regulatory effect on cardiac remodeling and cardiac fibrosis post‐MI.

#### Troponin‐I and cytokine measurements

**Figure 5:**
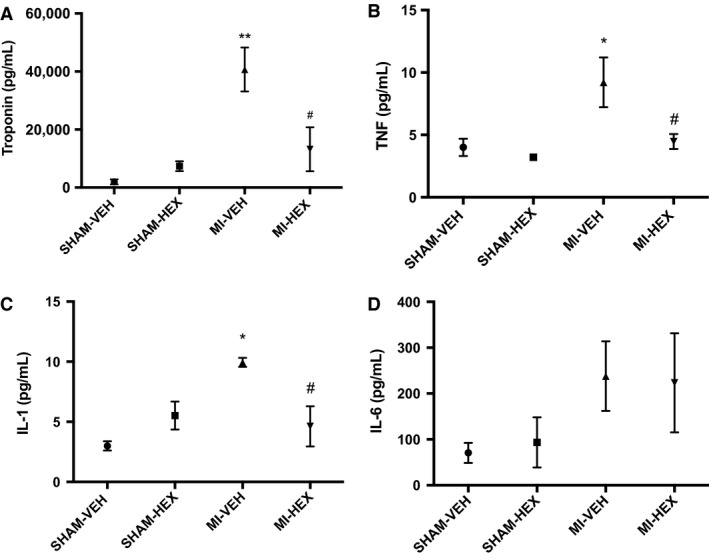
Serum assessment of: (A) Troponin‐I (pg/mL) (*n* = 4/4/5/5); (B) TNF‐*α* (pg/mL) (*n* = 4/4/4/4); (C) IL‐1*β* (pg/mL) (*n* = 3/3/4/5); (D) IL‐6 (pg/mL) (*n* = 4/4/5/4). Serum levels assessed 24 h post‐MI/sham procedure with or without HEX treatment. *P < 0.05, **P < 0.01, versus SHAM‐VEH group; ^#^ *P *< 0.05, versus MI‐VEH group.

There was a threefold increase in cTn‐I after 24 h in the MI‐VEH group compared with the MI‐HEX group (VEH 40,697 pg/mL ± SEM 7568; HEX 13,200 pg/mL ± SEM 7551). HEX treatment was found to significantly prevent the rise in cTnI (Fig. 5A). There was no significant difference in cTnI between the MI‐VEH and MI‐HEX group after 21 days but a trend toward a reduction in cTnI was maintained in the MI‐HEX group.

In MI‐VEH mice, serum TNF‐*α* (Fig. 5B) and IL‐1*β* (Fig. 5C) were significantly increased 24 h post‐MI compared to SHAM‐VEH counterparts and there was a trend toward increased IL‐6 levels (Fig. 5D). The acute MI‐induced aberrant increase in TNF‐*α* (VEH 9.94 pg/mL ± SEM 0.38; HEX 4.63 pg/mL ± SEM 1.6) (Fig. 5A) and IL‐1*β* (VEH 9.21 pg/mL ± SEM 2; HEX 4.47 pg/mL ± SEM 0.6) (Fig. 5B) was normalized by HEX (MI‐HEX) treatment, whereas IL‐6 levels were unaffected by HEX administration (Fig. 5C). In comparison, there were no differences noted in cytokine levels between MI‐HEX and MI‐VEH‐treatment at the 21‐day time point (data not shown).

#### Heart rate variability (HRV)

**Figure 6:**
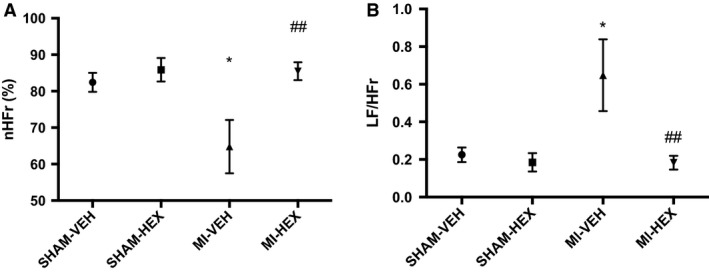
Heart rate variability analysis. Electrocardiographic data recorded 21 days post‐MI/sham procedure with or without HEX treatment. (A) Sympathetic nerve activity represented by low‐frequency power (LF/HFr), (*n* = 4/5/4/8). (B) Parasympathetic nerve activity represented by high‐frequency power (nHFr), (*n* = 4/5/4/8). *P < 0.05, versus SHAM‐VEH group; ^##^ *P *< 0.01, versus MI‐VEH group. HF, high frequency; LF, low frequency; nHF, normalized high‐frequency power.

HRV was examined 21 days after sham operation or permanent LAD occlusion. HRV analysis is considered a sensitive, noninvasive, indirect measure of cardiac autonomic tone that has been extensively validated (Task Force of the European Society of Cardiology 1996; Thireau et al. 2008). LF/HFr is used to represent cardiac sympathetic nervous activity (SNA), whereas nHFr represents parasympathetic nervous activity (PNA) (Thireau et al. 2008). LF/HFr was markedly increased (Fig. 6A), whereas nHFr was significantly reduced (Fig. 6B) in MI‐VEH mice compared to that measured from their SHAM‐VEH counterparts. HEX treatment (MI‐HEX group) normalized the MI‐induced loss of nHFr and increase in LF/HFr (Fig. 6) back to that measured in SHAM‐VEH mice, thereby indicating a simultaneous enhancement in PNA and reduction in sympathetic tone.

## Discussion

In this study, we have demonstrated that HEX treatment can preserve cardiac function and tissue characteristics in mice after acute MI. This was shown by an improvement in systolic and diastolic function, a lower cardiac mass and HW/TL ratio in MI‐injured mice after chronic HEX therapy. One of the major findings of this study was the ability of HEX to significantly reduce measures of cardiac fibrosis after MI. The antifibrotic properties of HEX may be associated with an underlying anti‐inflammatory mechanism, indicated in this study by a decrease in the proinflammatory cytokines TNF‐*α* and IL‐1*β* and significant reduction in cTnI levels. Furthermore, HEX treatment was seen to shift the balance of autonomic nervous system activity toward a parasympathetic predominance as evidenced by a lower LF/HFr ratio and higher nHFr measured by HRV analysis.

Our findings are consistent with those showing that HEX administration had markedly protected against myocardial stunning with almost complete recovery of LV function postreperfusion in senescent rats. A simultaneous reduction in creatine kinase concentration suggested a HEX‐mediated preservation of cardiomyocyte integrity (Rossoni et al. 1998). Locatelli et al. (1999), concluded that short‐term pretreatment with HEX counteracted ischemic damage in perfused hearts of hypophysectomized rats mediated through cardiac‐specific receptors and independent of GH‐release. At the clinical level, the effects of HEX were investigated in normal subjects and in patients with severe GH deficiency with similar conclusions (Imazio et al. 2002). Specifically, a similar increase in LVEF was seen after HEX treatment in normal subjects, patients with ischemic cardiomyopathy and those with severe GH deficiency, which was independent of GH‐release. Interestingly, in that study, acute IV administration of HEX had a positive inotropic effect in patients with ischemic cardiomyopathy‐induced severe LV dysfunction but failed to stimulate heart contractility in patients with severe LV dysfunction due to dilated cardiomyopathy (Imazio et al. 2002).

### Cardiac fibrosis and remodeling

**Figure 7:**
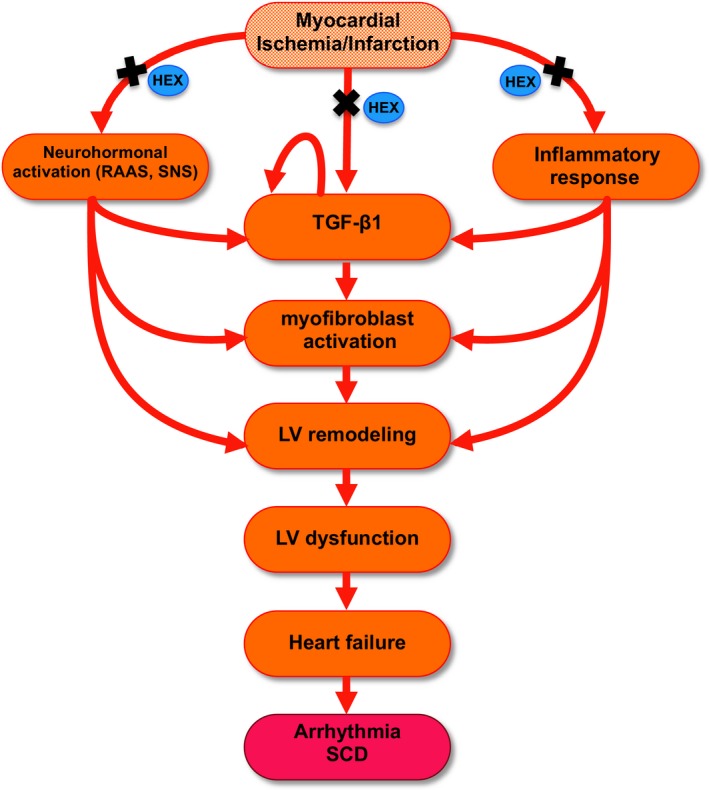
Hexarelin: proposed mechanisms of action (see text). MI induces dynamic alterations in fibroblast phenotype, initiates an inflammatory response and activates neurohormonal pathways that collectively result in cardiac remodeling, mechanical dysfunction, and electrical disturbances in the failing heart, increasing the risk of sudden cardiac death (Dixon et al. 2015). TFG‐*β*1 is markedly augmented following MI and is the most potent profibrotic cytokine known, causing excessive extracellular matrix production, inducing its own secretion and driving myofibroblast activation (Dixon et al. 2015). Inflammatory cytokines exert potent proinflammatory actions on cardiac fibroblasts and can negatively affect LV function. Similarly, the RAAS & SNA play a critical role in activation of myofibroblasts and influence LV remodeling & dysfunction (Sun and Weber 2000; Dixon et al. 2015). HEX may attenuate and actively reverse cardiac remodeling and myocardial fibrosis by targeting these pathways (indicated by “x”) and thus holds promise in returning the failing heart to a functional state. RAAS, renin angiotensin aldosterone system; SNA, sympathetic nervous activation.

After myocardial injury, multiple neurohormonal factors and cytokines drive changes in cardiomyocytes and fibroblasts that collectively result in cardiac remodeling (Dixon et al. 2015) (Fig. 7). MI leads to progressive ventricular remodeling, increased myocardial wall stress and ultimately results in HF (Huang et al. 2009). During ventricular remodeling, collagen, the main component of the extracellular matrix (ECM) increases and contributes to scar tissue (fibrosis) formation. When increases in wall stress exceed that of the compensatory capacity of the heart, degradation of the extracellular matrix and alterations of the collagen network progressively result in alterations of LV morphology and function. Continued accumulation of collagen (fibrosis) impairs diastolic function and compromises systolic mechanisms (Segura et al. 2014).

The antifibrotic effects of GHS have been investigated in various models of cardiac injury (Mao et al. 2014a; Angelino et al. 2015). Interventions targeting excessive fibrosis as a result of MI are of major therapeutic importance in the treatment of HF (Samuel et al. 2011; Segura et al. 2014). HEX treatment may allow normalization of wall stress and preservation of diastolic and systolic function, helping to prevent changes in ventricular wall and chamber dimensions. In this study, HEX therapy blunted changes in LV mass and significantly reduced interstitial LV collagen content and collagen concentration. These findings represent the therapeutic potential of HEX to alleviate degenerative hypertrophic adaptive changes leading to LV dysfunction.

### Inflammation and remodeling

HEX has been shown to inhibit apoptosis, increase cardiomyocyte viability and suppress stress‐induced neurohormonal activation in various studies (Filigheddu et al. 2001; Xu et al. 2005; Zhao et al. 2016). HEX has been demonstrated to protect cardiomyocytes against angiotensin (AT)‐II‐induced apoptosis by inhibiting activation of caspase‐3, Bax mRNA expression and increasing the expression of Bcl‐2 (Pang et al. 2004). In addition to inhibition of apoptosis, anti‐inflammatory strategies may represent another therapeutic approach to mitigate cardiac fibrosis. Timely activation of the endogenous pathways that inhibit inflammation is important to prevent the catastrophic consequences of uncontrolled inflammation on cardiac geometry and function post myocardial infarction (Dixon et al. 2015). Induction of proinflammatory cytokines, such as IL‐1 and TNF‐*α* play an important role in acquisition of a proinflammatory phenotype by cardiac fibroblasts during the early stages following MI and have been demonstrated to play a role in cardiac remodeling and progression of HF (Saxena et al. 2013). This study demonstrated the ability of HEX to reduce the presence of serum IL‐1*β* and TNF‐*α* in a model of MI (Fig. 7).

Sustained presence of inflammatory cytokines leads to activation of MMPs and results in ventricular dilation through slippage of collagen (Bryant‐Greenwood 1991; Deschamps and Spinale 2006). Our data suggest that HEX may increase the degradation of collagen through increased MMP activity demonstrated by an increase in MMP‐13 expression after chronic HEX treatment. Xu et al. (2012) also observed similar findings in a study where spontaneously hypertensive rats were treated with HEX. In this study, treatment resulted in a significant reduction in cardiac fibrosis, attenuation of LV hypertrophy, diastolic dysfunction, and reduced blood pressure. HEX increased MMP‐2 and MMP‐9 activity and decreased myocardial mRNA expression of TIMP1 suggesting an increased degradation of collagen. Thus, we propose that HEX may reverse the mechanisms that lead to progressive LV dysfunction, at least in part, through inhibition of the inflammatory response and regulation of collagen turnover.

### Myofibroblast activation

Compared with cardiomyocytes, cardiac fibroblasts are much less vulnerable to oxidant stress following coronary occlusion (Zhang et al. 2001). Given their resistance to ischemic death, location and abundance within the cardiac interstitium, fibroblasts are ideally suited to sense microenvironmental alterations following myocardial injury and initiate the inflammatory response (Chen and Frangogiannis 2013). In vitro, cardiac fibroblasts respond to hypoxia by acquiring a proinflammatory and fibrogenic phenotype characterized by enhanced cytokine expression, myofibroblast transdifferentiation, and increased collagen synthesis (Shivakumar et al. 2008). Experimental findings suggest a direct involvement of fibroblasts in activation of the postinfarction inflammatory reaction (Chen and Frangogiannis 2013). TGF‐*β*1 expression is markedly augmented following infarction and has been found to have profound effects on fibroblast phenotype and function, inducing myofibroblast transdifferentiation, upregulating matrix protein synthesis and stimulating the synthesis of protease inhibitors such as TIMP‐1 (Willems et al. 1994; Dixon et al. 2015). Our study demonstrated a significant reduction in *α*‐SMA expression in HEX‐treated mice in combination with a decrease in TGF‐*β*1. This suggests decreased activation of myofibroblasts secondary to a HEX‐mediated reduction in TGF‐*β*1 and inflammatory cytokines. Preventing TGF‐*β*1 activation in a myofibroblast‐specific manner has promising therapeutic perspectives and HEX may represent a novel pharmacological agent to target this pathway.

Extensive evidence also suggests a crucial role of the renin‐angiotensin aldosterone (RAAS) system in activation of infarct myofibroblasts (Dixon et al. 2015). The fibrogenic effects of the RAAS are mediated, at least in part, through activation of growth factors such as TGF‐*β*1 (Detillieux et al. 2003). HEX has been shown to suppress ATII and TGF‐*β*1‐induced cardiac fibroblast proliferation and collagen synthesis in cultured rat cardiac fibroblasts (Xu et al. 2007). Our study demonstrated a significant reduction in TGF‐*β*1 expression in HEX‐treated mice. Thus suggesting that HEX's ability to influence postinfarct fibrotic healing may involve the ability to suppress the expression and effect of profibrotic factors (such as TGF‐*β*1), that promote fibrogenesis; inhibit myofibroblast differentiation and myofibroblast‐induced collagen synthesis; and augment MMP‐13‐induced collagen breakdown. The combined actions of which favor a net reduction in aberrant collagen content.

### Autonomic nervous system

It is well‐known that activation of efferent vagal nerve fibers can modulate local and systemic inflammatory responses through activation of the “cholinergic anti‐inflammatory pathway” (Tracey 2007). The vagus is suggested to play a central role in GHS‐observed effects and is an important link between the nervous and immune systems. Ghrelin has been shown to have several anti‐inflammatory properties involving vagus activation (Date et al. 2002; Mao et al. 2014a; Khowailed et al. 2015). The restoration of parasympathetic tone has emerged as a promising therapeutic approach to normalize autonomic imbalance and inhibit the progression of HF (Kaye et al. 1995; Olshansky 2016). Furthermore, SNA in patients with MI has been shown to contribute significantly to disease progression and prognosis (Kaye et al. 1995). Accumulating clinical and experimental evidence indicates that inhibition of cardiac SNA improves survival and mitigates LV remodeling and dysfunction post‐MI (Schwenke et al. 2008, 2012). In this study, chronic HEX treatment was demonstrated to modulate the autonomic nervous system by blunting SNA and enhancing PSA, and influence the inflammatory response induced by MI.

Given that the foremost role of ghrelin relates to the control of systemic metabolism, one possible mechanism underlying the beneficial effect of ghrelin in heart failure may relate to normalization of cardiac energy substrate consumption and attenuation of cardiac metabolic disturbances (Xu et al. 2010; Mitacchione et al. 2014). These pathways may also involve modulation of the autonomic nervous system (Flaa et al. 2008).

### Limitations

HEX administration immediately prior to ligation represents a potential limitation. Timely reperfusion strategies act to limit infarct development, thus more logically; therapy would be initiated at this point, prior to reperfusion. The effect of reperfusion was not investigated in this study and represents a downfall. The temporal histopathological and functional infarct changes in mouse models of MI vary when compared to acute myocardial infarction patients, thus representing a limitation of this model.

## Conclusion

In this study, we have demonstrated that HEX treatment has a clear effect on myocardial function, fibrosis, and inflammation in a mouse model of MI. Pharmacological strategies targeting fibrogenic growth factors and anti‐inflammatory effects may hold promise in prevention of cardiac remodeling and attenuation of diastolic and systolic dysfunction. Modulation of the autonomic nervous system by HEX may offer a novel cardioprotective strategy by acting to modulate the inflammatory reaction and fibrotic pathways.

## Conflict of Interest

None declared.

## References

[phy213699-bib-0001] Angelino, E. , S. Reano , M. Ferrara , E. Agosti , A. Graziani , and N. Filigheddu . 2015 Antifibrotic activity of acylated and unacylated ghrelin. Int. J. Endocrinol. 2015:385682. 25960743 10.1155/2015/385682 PMC4415458

[phy213699-bib-0002] Baldanzi, G. , N. Filigheddu , S. Cutrupi , F. Catapano , S. Bonissoni , A. Fubini , et al. 2002 Ghrelin and des‐acyl ghrelin inhibit cell death in cardiomyocytes and endothelial cells through ERK1/2 and PI 3‐kinase/AKT. J. Cell Biol. 159:1029–1037. 12486113 10.1083/jcb.200207165 PMC2173981

[phy213699-bib-0003] Bohl, S. , C. A. Lygate , H. Barnes , D. Medway , L. A. Stork , J. Schulz‐Menger , et al. 2009 Advanced methods for quantification of infarct size in mice using three‐dimensional high‐field late gadolinium enhancement MRI. Am. J. Physiol. Heart Circ. Physiol. 296:H1200–H1208. 19218501 10.1152/ajpheart.01294.2008 PMC2670705

[phy213699-bib-0004] Bovens, S. M. , B. Boekhorst , K. den Ouden , K. W. A. van de Kolk , A. Nauerth , M. G. J. Nederhoff , et al. 2011 Evaluation of infarcted murine heart function: comparison of prospectively triggered with self‐gated MRI. NMR Biomed. 24:307–315. 20891021 10.1002/nbm.1593

[phy213699-bib-0005] Bryant‐Greenwood, G. D. 1991 Human decidual and placental relaxins. Reprod. Fertil. Dev. 3:385–389. 1957026 10.1071/rd9910385

[phy213699-bib-0006] Chen, W. , and N. G. Frangogiannis . 2013 Fibroblasts in post‐infarction inflammation and cardiac repair. Biochim. Biophys. Acta 1833:945–953. 22982064 10.1016/j.bbamcr.2012.08.023 PMC3541439

[phy213699-bib-0007] Cheng, K. C. , Y. X. Li , A. Asakawa , and A. Inui . 2010 The role of ghrelin in energy homeostasis and its potential clinical relevance. Int. J. Mol. Med. 26(6):771–778. 21042769 10.3892/ijmm_00000524

[phy213699-bib-0008] Date, Y. , M. Kojima , H. Hosoda , A. Sawaguchi , M. S. Mondal , T. Suganuma , et al. 2000 Ghrelin, a novel growth hormone‐releasing acylated peptide, is synthesized in a distinct endocrine cell type in the gastrointestinal tracts of rats and humans 1. Endocrinology 141:4255–4261. 11089560 10.1210/endo.141.11.7757

[phy213699-bib-0009] Date, Y. , N. Murakami , K. Toshinai , S. Matsukura , A. Niijima , H. Matsuo , et al. 2002 The role of the gastric afferent vagal nerve in ghrelin‐induced feeding and growth hormone secretion in rats. Gastroenterology 123:1120–1128. 12360474 10.1053/gast.2002.35954

[phy213699-bib-0010] Deghenghi, R. 1998 Synthetic peptides and their non‐peptidyl mimetics in endocrinology: from synthesis to clinical perspectives. J. Endocrinol. Invest. 21:787–793. 9972682 10.1007/BF03348048

[phy213699-bib-0011] Deschamps, A. M. , and F. G. Spinale . 2006 Pathways of matrix metalloproteinase induction in heart failure: bioactive molecules and transcriptional regulation. Cardiovasc. Res. 69:666–676. 16426590 10.1016/j.cardiores.2005.10.004

[phy213699-bib-0012] Detillieux, K. A. , F. Sheikh , E. Kardami , and P. A. Cattini . 2003 Biological activities of fibroblast growth factor‐2 in the adult myocardium. Cardiovasc. Res. 57:8–19. 12504809 10.1016/s0008-6363(02)00708-3

[phy213699-bib-0013] Dixon, I. M. , R. H. Cunnington , S. G. Rattan , and J. T. Wigle . 2015 Cardiac fibrosis and heart failure—cause or effect? Pp. 1–4 *in* Dixon I. and Wigle J. T., eds. Cardiac fibrosis and heart failure: cause or effect?. Springer, Cham.

[phy213699-bib-0014] Filigheddu, N. , A. Fubini , G. Baldanzi , S. Cutrupi , C. Ghè , F. Catapano , et al. 2001 Hexarelin protects H9c2 cardiomyocytes from doxorubicin‐induced cell death. Endocrine 14:113–119. 11322493 10.1385/ENDO:14:1:113

[phy213699-bib-0015] Flaa, A. , T. A. Aksnes , S. E. Kjeldsen , I. Eide , and M. Rostrup . 2008 Increased sympathetic reactivity may predict insulin resistance: an 18‐year follow‐up study. Metabolism 57:1422–1427. 18803948 10.1016/j.metabol.2008.05.012

[phy213699-bib-0016] Gallop, P. M. , and M. A. Paz . 1975 Posttranslational protein modifications, with special attention to collagen and elastin. Physiol. Rev. 55:418–487. 50603 10.1152/physrev.1975.55.3.418

[phy213699-bib-0017] Guan, X.‐M. , H. Yu , O. C. Palyha , K. K. McKee , S. D. Feighner , D. J. Sirinathsinghji , et al. 1997 Distribution of mRNA encoding the growth hormone secretagogue receptor in brain and peripheral tissues. Mol. Brain Res. 48:23–29. 9379845 10.1016/s0169-328x(97)00071-5

[phy213699-bib-0018] Hausenloy, D. J. , and D. M. Yellon . 2013 Myocardial ischemia‐reperfusion injury: a neglected therapeutic target. J. Clin. Invest. 123:92–100. 23281415 10.1172/JCI62874 PMC3533275

[phy213699-bib-0019] Huang, C. X. , M. J. Yuan , H. Huang , G. Wu , Y. Liu , S. B. Yu , et al. 2009 Ghrelin inhibits post‐infarct myocardial remodeling and improves cardiac function through anti‐inflammation effect. Peptides 30:2286–2291. 19747956 10.1016/j.peptides.2009.09.004

[phy213699-bib-0020] Imazio, M. , M. Bobbio , F. Broglio , A. Benso , V. Podio , M. Valetto , et al. 2002 GH‐independent cardiotropic activities of hexarelin in patients with severe left ventricular dysfunction due to dilated and ischemic cardiomyopathy. Eur. J. Heart Fail. 4:185–191. 11959048 10.1016/s1388-9842(01)00223-9

[phy213699-bib-0021] Kaye, D. M. , J. Lefkovits , G. L. Jennings , P. Bergin , A. Broughton , and M. D. Esler . 1995 Adverse consequences of high sympathetic nervous activity in the failing human heart. J. Am. Coll. Cardiol. 26:1257–1263. 7594040 10.1016/0735-1097(95)00332-0

[phy213699-bib-0022] Khowailed, A. , S. M. Younan , H. Ashour , A. E. Kamel , and N. Sharawy . 2015 Effects of ghrelin on sepsis‐induced acute kidney injury: one step forward. Clin. Exp. Nephrol. 19:419–426. 25002019 10.1007/s10157-014-1006-x

[phy213699-bib-0023] Kishimoto, I. , T. Tokudome , D. O. Schwenke , S. Takeshi , H. Hosoda , N. Nagaya , et al. 2009 Therapeutic potential of ghrelin in cardiac diseases. Expert Rev. Endocrinol. Metab. 4:283–289. 10.1586/eem.09.7 30743789

[phy213699-bib-0024] Kojima, M. , and K. Kangawa . 2005 Ghrelin: structure and function. Physiol. Rev. 85:495–522. 15788704 10.1152/physrev.00012.2004

[phy213699-bib-0025] Kojima, M. , H. Hosoda , Y. Date , M. Nakazato , H. Matsuo , and K. Kangawa . 1999 Ghrelin is a growth‐hormone‐releasing acylated peptide from stomach. Nature 402:656–660. 10604470 10.1038/45230

[phy213699-bib-0026] Locatelli, V. , G. Rossoni , F. Schweiger , A. Torsello , Colonna V. De Gennaro , M. Bernareggi , et al. 1999 Growth hormone‐independent cardioprotective effects of hexarelin in the rat. Endocrinology 140:4024–4031. 10465272 10.1210/endo.140.9.6948

[phy213699-bib-0027] Mao, Y. , T. Tokudome , K. Otani , I. Kishimoto , M. Nakanishi , H. Hosoda , et al. 2012 Ghrelin prevents incidence of malignant arrhythmia after acute myocardial infarction through vagal afferent nerves. Endocrinology 153:3426–3434. 22535766 10.1210/en.2012-1065

[phy213699-bib-0028] Mao, Y. , T. Tokudome , I. Kishimoto , K. Otani , H. Hosoda , C. Nagai , et al. 2013 Hexarelin treatment in male ghrelin knockout mice after myocardial infarction. Endocrinology 154:3847–3854. 23861368 10.1210/en.2013-1291

[phy213699-bib-0029] Mao, Y. , T. Tokudome , and I. Kishimoto . 2014a The cardiovascular action of hexarelin. J. Geriatr. Cardiol. 11:253–258. 25278975 10.11909/j.issn.1671-5411.2014.03.007 PMC4178518

[phy213699-bib-0030] Mao, Y. , T. Tokudome , I. Kishimoto , K. Otani , M. Miyazato , and K. Kangawa . 2014b One dose of oral hexarelin protects chronic cardiac function after myocardial infarction. Peptides 56:156–162. 24747279 10.1016/j.peptides.2014.04.004

[phy213699-bib-0031] Mitacchione, G. , J. C. Powers , G. Grifoni , F. Woitek , A. Lam , L. Ly , et al. 2014 The gut hormone ghrelin partially reverses energy substrate metabolic alterations in the failing heart. Circ. Heart Fail. 7:643–651. 24855152 10.1161/CIRCHEARTFAILURE.114.001167 PMC4118426

[phy213699-bib-0032] Mori, K. , A. Yoshimoto , K. Takaya , K. Hosoda , H. Ariyasu , K. Yahata , et al. 2000 Kidney produces a novel acylated peptide, ghrelin. FEBS Lett. 486:213–216. 11119706 10.1016/s0014-5793(00)02308-5

[phy213699-bib-0033] Nagaya, N. , M. Kojima , M. Uematsu , M. Yamagishi , H. Hosoda , H. Oya , et al. 2001 Hemodynamic and hormonal effects of human ghrelin in healthy volunteers. Am. J. Physiol. Regul. Integr. Comp. Physiol. 280:R1483–R1487. 11294772 10.1152/ajpregu.2001.280.5.R1483

[phy213699-bib-0034] Olshansky, B. 2016 Vagus nerve modulation of inflammation: cardiovascular implications. Trends Cardiovasc. Med. 26:1–11. 25939778 10.1016/j.tcm.2015.03.016

[phy213699-bib-0035] Pang, J.‐J. , R.‐K. Xu , X.‐B. Xu , J.‐M. Cao , C. Ni , W.‐L. Zhu , et al. 2004 Hexarelin protects rat cardiomyocytes from angiotensin II‐induced apoptosis in vitro. Am. J. Physiol. Heart Circ. Physiol. 286:H1063–H1069. 14615277 10.1152/ajpheart.00648.2003

[phy213699-bib-0036] Rosset, A. , L. Spadola , and O. Ratib . 2004 OsiriX: an open‐source software for navigating in multidimensional DICOM images. J. Digit. Imaging 17:205–216. 15534753 10.1007/s10278-004-1014-6 PMC3046608

[phy213699-bib-0037] Rossoni, G. , V. D. G. Colonna , M. Bernareggi , G. L. Polvani , E. E. Müller , and F. Berti . 1998 Protectant activity of hexarelin or growth hormone against postischemic ventricular dysfunction in hearts from aged rats. J. Cardiovasc. Pharmacol. 32:260–265. 9700988 10.1097/00005344-199808000-00013

[phy213699-bib-0038] Samuel, C. S. 2009 Determination of collagen content, concentration, and sub‐types in kidney tissue. Methods Mol. Biol. 466:223–235. 19148607 10.1007/978-1-59745-352-3_16

[phy213699-bib-0039] Samuel, C. S. , S. Cendrawan , X. M. Gao , Z. Ming , C. Zhao , H. Kiriazis , et al. 2011 Relaxin remodels fibrotic healing following myocardial infarction. Lab. Invest. 91:675–690. 21221074 10.1038/labinvest.2010.198

[phy213699-bib-0040] Saxena, A. , W. Chen , Y. Su , V. Rai , O. U. Uche , N. Li , et al. 2013 IL‐1 induces proinflammatory leukocyte infiltration and regulates fibroblast phenotype in the infarcted myocardium. J. Immunol. 191:4838–4848. 24078695 10.4049/jimmunol.1300725 PMC3822582

[phy213699-bib-0041] Schneider, C. A. , W. S. Rasband , and K. W. Eliceiri . 2012 NIH Image to ImageJ: 25 years of image analysis. Nat. Methods 9:671–675. 22930834 10.1038/nmeth.2089 PMC5554542

[phy213699-bib-0042] Schwenke, D. O. , T. Tokudome , I. Kishimoto , T. Horio , M. Shirai , P. A. Cragg , et al. 2008 Early ghrelin treatment after myocardial infarction prevents an increase in cardiac sympathetic tone and reduces mortality. Endocrinology 149:5172–5176. 18599547 10.1210/en.2008-0472

[phy213699-bib-0043] Schwenke, D. O. , T. Tokudome , I. Kishimoto , T. Horio , P. A. Cragg , M. Shirai , et al. 2012 One dose of ghrelin prevents the acute and sustained increase in cardiac sympathetic tone after myocardial infarction. Endocrinology 153:2436–2443. 22434083 10.1210/en.2011-2057

[phy213699-bib-0044] Segura, A. M. , O. H. Frazier , and L. M. Buja . 2014 Fibrosis and heart failure. Heart Fail. Rev. 19:173–185. 23124941 10.1007/s10741-012-9365-4

[phy213699-bib-0045] Shivakumar, K. , S. J. Sollott , M. Sangeetha , S. Sapna , B. Ziman , S. Wang , et al. 2008 Paracrine effects of hypoxic fibroblast‐derived factors on the MPT‐ROS threshold and viability of adult rat cardiac myocytes. Am. J. Physiol. Heart Circ. Physiol. 294:H2653–H2658. 18408121 10.1152/ajpheart.91443.2007 PMC5875700

[phy213699-bib-0046] Sun, Y. , and K. T. Weber . 2000 Infarct scar: a dynamic tissue. Cardiovasc. Res. 46:250–256. 10773228 10.1016/s0008-6363(00)00032-8

[phy213699-bib-0047] Suvarna, S . (2013). Cardiac pathology: a guide to current practice. Springer Science & Business Media, Berlin, Germany.

[phy213699-bib-0048] Takagawa, J. , Y. Zhang , M. L. Wong , R. E. Sievers , N. K. Kapasi , Y. Wang , et al. 2007 Myocardial infarct size measurement in the mouse chronic infarction model: comparison of area‐ and length‐based approaches. J. Appl. Physiol. 1985(102):2104–2111. 10.1152/japplphysiol.00033.2007 PMC2675697 17347379

[phy213699-bib-0049] Tarnavski, O. , J. R. McMullen , M. Schinke , Q. Nie , S. Kong , and S. Izumo . 2004 Mouse cardiac surgery: comprehensive techniques for the generation of mouse models of human diseases and their application for genomic studies. Physiol. Genomics 16:349–360. 14679301 10.1152/physiolgenomics.00041.2003

[phy213699-bib-0050] Task Force of the European Society of Cardiology . (1996). Heart rate variability standards of measurement, physiological interpretation, and clinical use. Eur. Heart J. 17, 354–381. 8737210

[phy213699-bib-0051] Thireau, J. , B. L. Zhang , D. Poisson , and D. Babuty . 2008 Heart rate variability in mice: a theoretical and practical guide. Exp. Physiol. 93:83–94. 17911354 10.1113/expphysiol.2007.040733

[phy213699-bib-0052] Tracey, K. J. 2007 Physiology and immunology of the cholinergic antiinflammatory pathway. J. Clin. Invest. 117:289–296. 17273548 10.1172/JCI30555 PMC1783813

[phy213699-bib-0053] Willems, I. , M. G. Havenith , J. De Mey , and M. Daemen . 1994 The alpha‐smooth muscle actin‐positive cells in healing human myocardial scars. Am. J. Pathol. 145:868. 7943177 PMC1887334

[phy213699-bib-0054] Xu, X. B. , J. J. Pang , J. M. Cao , C. Ni , R. K. Xu , X. Z. Peng , et al. 2005 GH‐releasing peptides improve cardiac dysfunction and cachexia and suppress stress‐related hormones and cardiomyocyte apoptosis in rats with heart failure. Am. J. Physiol. Heart Circ. Physiol. 289:H1643–H1651. 15951341 10.1152/ajpheart.01042.2004

[phy213699-bib-0055] Xu, X. , J. Pang , H. Yin , M. Li , W. Hao , C. Chen , et al. 2007 Hexarelin suppresses cardiac fibroblast proliferation and collagen synthesis in rat. Am. J. Physiol. Heart Circ. Physiol. 293:H2952–H2958. 17766487 10.1152/ajpheart.00004.2007

[phy213699-bib-0056] Xu, J. P. , H. X. Wang , W. Wang , L. K. Zhang , and C. S. Tang . 2010 Ghrelin improves disturbed myocardial energy metabolism in rats with heart failure induced by isoproterenol. J. Pept. Sci. 16:392–402. 20572026 10.1002/psc.1253

[phy213699-bib-0057] Xu, X. , F. Ding , J. Pang , X. Gao , R. K. Xu , W. Hao , et al. 2012 Chronic administration of hexarelin attenuates cardiac fibrosis in the spontaneously hypertensive rat. Am. J. Physiol. Heart Circ. Physiol. 303:H703–H711. 22842067 10.1152/ajpheart.00257.2011

[phy213699-bib-0058] Zhang, X. , G. Azhar , K. Nagano , and J. Y. Wei . 2001 Differential vulnerability to oxidative stress in rat cardiac myocytes versus fibroblasts. J. Am. Coll. Cardiol. 38:2055–2062. 11738315 10.1016/s0735-1097(01)01665-5

[phy213699-bib-0059] Zhao, Y. , X. Zhang , J. Chen , C. Lin , R. Shao , C. Yan , et al. 2016 Hexarelin protects rodent pancreatic Β‐cells function from cytotoxic effects of streptozotocin involving mitochondrial signalling pathways in vivo and in vitro. PLoS ONE 11:e0149730. 26918825 10.1371/journal.pone.0149730 PMC4769129

